# Sexuality in the Elderly: Challenges and Opportunities

**DOI:** 10.1192/j.eurpsy.2025.884

**Published:** 2025-08-26

**Authors:** F. G. Tavares, B. C. Araújo, B. T. Wildenberg, P. M. Esteves, L. Carrasqueira, J. A. Brás, M. Coroa

**Affiliations:** 1 Psychiatry, ULS Algarve, Faro; 2 Psychiatry, ULS Coimbra, Coimbra; 3 Psychiatry, ULS Região Leiria, Leiria, Portugal

## Abstract

**Introduction:**

Sexuality in the elderly has become an increasingly important topic in healthcare as the global population continues to age, raising new challenges and considerations related to the quality of life and well-being of older adults.

**Objectives:**

This work aims to explore the barriers faced by the elderly regarding sexuality and propose strategies for promoting healthy and fulfilling sexuality at this stage of life.

**Methods:**

A narrative review was employed on the topic, aiming to broadly and exploratorily understand the main aspects related to sexuality in the elderly population.

**Results:**

Recent studies suggest that sexual activity can remain an important part of life for older individuals, positively influencing both mental and physical health. The main obstacles to healthy sexuality in old age can be broadly categorized into physiological, psychological, and sociocultural factors. **Physiological changes** include a natural decline in hormone levels, such as estrogen in women and testosterone in men, leading to reduced libido, vaginal dryness, and erectile dysfunction. Chronic illnesses like cardiovascular disease, diabetes, and arthritis, along with medications for these conditions, can further impact sexual function. **Psychological factors**, such as anxiety, depression, and reduced self-esteem due to aging-related body changes, also play a significant role in diminishing sexual desire and activity. **Sociocultural factors** include long-standing societal taboos around older adults and sexuality, which can lead to embarrassment, reluctance to discuss sexual health issues, and feelings of shame. Healthcare professionals can adopt several strategies to improve sexuality in aging such as o**pen communication.** Regular sexual health assessments should be integrated into routine care, including questions about sexual function, relationship satisfaction, and any challenges faced. **Medical interventions**, such as hormone replacement therapy or treatments for erectile dysfunction can address physiological barriers. **Psychosocial support** can improve communication, body image issues, and mental health factors like anxiety or depression that often accompany aging.

**Conclusions:**

The approach to sexuality in the elderly should be multifaceted, integrating biopsychosocial perspectives, with an emphasis on promoting sexual education and providing appropriate treatments that address individual challenges. Healthcare professionals shouldadopt a welcoming and open attitude, encouraging dialogue on this topic to improve the quality of life of older adults.

**Disclosure of Interest:**

None Declared

